# Emotional Intelligence and Language Learning Performance of ESL Learners: Mediating Effects of L2 Grit and L2 Motivation

**DOI:** 10.3390/jintelligence13110146

**Published:** 2025-11-13

**Authors:** Qingshu Xu, Huaqing Hong

**Affiliations:** 1 School of Aeronautics, Shandong Jiaotong University, Jinan 250357, China; qsxu1987@umd.edu; 2 College of Education, University of Maryland, College Park, MD 20742, USA; 3 Institute of Language Sciences, Shanghai International Studies University, Shanghai 201620, China; 4 Center for Research and Development in Learning, Nanyang Technological University, Singapore 637335, Singapore

**Keywords:** emotional intelligence, L2 grit, L2 motivation, chain mediation, ESL performance, structural equation modeling

## Abstract

This study examined the associations among emotional intelligence (EI), grit, motivation, and second language (L2) learning performance, with particular attention to the mediating roles of grit and motivation. A sample of 801 Chinese university students completed validated measures of EI, grit, and motivation, and their English test scores were collected as an indicator of performance. Using structural equation modeling (SEM), EI was positively associated with grit (β = 0.574, *p* < .001) and motivation (β = 0.426, *p* < .001), while grit was also positively related to motivation (β = 0.515, *p* < .001). In relation to performance, significant positive associations were observed with motivation (β = 0.635, *p* < .001), EI (β = 0.185, *p* < .001), and grit (β = 0.165, *p* < .001). Bootstrap analyses confirmed robust indirect associations of EI with performance through grit (β = 0.095), through motivation (β = 0.271), and via the sequential chain EI → grit → motivation → performance (β = 0.188). The model accounted for 33% of the variance in grit, 55% in motivation, and 79% in performance. These findings indicate that EI, grit, and motivation are closely interrelated constructs that jointly contribute to L2 performance, highlighting the importance of socio-emotional and motivational resources in second language learning.

## 1. Introduction

Learning a second or foreign language is not merely a linguistic endeavor but a cognitively demanding and emotionally dynamic process that requires sustained regulation of attention, effort, and affective states. In English-as-a-second-language (ESL) contexts, learners must cope with high cognitive load, competitive assessments, and limited opportunities for authentic communication, all of which contribute to heightened emotional strain and motivational fluctuations (Dörnyei and Ryan 2015; Li et al. 2025). While traditional research in second language acquisition (SLA) has primarily emphasized cognitive and linguistic predictors of success, an increasing body of work has recognized that non-cognitive factors—such as emotions, perseverance, and motivation—play equally crucial roles in shaping learning trajectories (MacIntyre and Gregersen 2012). However, despite this growing attention, existing studies often examine these factors in isolation, leaving unclear how emotional and motivational processes interact to influence sustained language learning performance. Addressing this gap requires a theoretically integrated model that elucidates emotional competence, effortful persistence, and motivational engagement within a coherent explanatory framework.

Among these non-cognitive factors, emotional intelligence (EI) has emerged as a key construct in applied linguistics and educational psychology. Defined by Salovey and Mayer (1990) as the ability to perceive, understand, regulate, and utilize emotions in oneself and others, EI has been conceptualized under two major frameworks: ability EI, which reflects actual emotional problem-solving abilities measured through performance-based tasks, and trait EI, which represents individuals’ self-perceived emotional efficacy and dispositions, typically assessed through self-report questionnaires (Pérez et al. 2005; Petrides and Furnham 2000). The present study adopts the trait EI perspective and employs the Wong and Law Emotional Intelligence Scale (WLEIS; Wong and Law 2002), a validated self-report measure widely used in educational settings. This choice aligns with the study’s focus on learners’ perceived emotional regulation and self-appraisal abilities as predictors of motivation and performance—domains that are best captured through subjective emotional awareness rather than objective ability testing. This conceptualization follows the methodological distinctions outlined by Petrides and Furnham (2000) and Joseph and Newman (2010), who emphasized that trait EI reflects affective self-perceptions integrated within personality hierarchies, whereas ability EI captures maximal performance on emotion-related cognitive tasks. Empirical evidence has consistently shown that higher trait EI is associated with reduced language anxiety, stronger self-regulation, and enhanced academic achievement (Dewaele 2019; Li et al. 2024; Jin et al. 2024). These findings suggest that learners’ emotional competencies not only influence how they experience the affective dimension of language learning but also shape the motivational processes that sustain their engagement over time.

Another important set of constructs that bridge emotion and achievement are L2 grit and L2 motivation. Grit, defined as the perseverance and sustained passion for long-term language learning goals (Duckworth et al. 2007; Teimouri et al. 2022), enables learners to maintain consistent effort despite setbacks and fluctuations in external reinforcement. Motivation, on the other hand, reflects the goal-directed energy that activates and sustains engagement in learning (Dörnyei 2005; Ryan and Deci 2000). Although both constructs are positively correlated, research suggests that grit and motivation operate at different temporal and functional levels: grit reflects the endurance to persist, whereas motivation captures the dynamic intensity of engagement at a given moment (Credé et al. 2017; Datu et al. 2016). Emotionally intelligent learners—who can accurately perceive and regulate emotions—are more likely to maintain a sense of control and positive value toward learning tasks, thereby enhancing perseverance (grit) and then sustaining motivation (Gao 2025; Chang and Tsai 2022).

The Control–Value Theory (Pekrun 2006; Pekrun and Perry 2014) provides a theoretical rationale for this sequence, positing that emotions influence learners’ perceived control and value appraisals, which in turn sustain perseverance (grit) and fuel motivation. From this perspective, emotionally intelligent learners are better able to regulate negative emotions such as anxiety and to maintain long-term commitment, which subsequently reinforces motivational intensity and engagement (Teimouri et al. 2022; Gao 2025). The L2 Motivational Self System (Dörnyei 2009) similarly supports this temporal ordering, as the ability to persevere toward one’s ideal L2 self-fosters sustained motivation to achieve it. Therefore, in the present study, grit is conceptualized as a precursor of motivation, representing the self-regulatory stamina that enables learners to translate emotional resources into enduring motivational engagement over time. EI may not only exert direct effects on language performance but also indirectly influence achievement through the sequential pathway of grit and motivation.

However, while previous studies have separately examined the effects of EI, grit, and motivation on language outcomes, few have empirically tested their combined mediating mechanisms within a single model, particularly in the Chinese ESL context (Peng and Li 2025). To address this gap, the present study investigates how Chinese ESL learners’ emotional intelligence (EI) relates to their language learning performance through the sequential mediation of L2 grit and L2 motivation. By integrating these three constructs into a chain mediation framework, the study aims to clarify the emotional–motivational mechanisms that underlie language achievement. Specifically, this research makes three verifiable contributions. Firstly, at the theoretical level, it extends the application of the Control–Value Theory (Pekrun 2006) and L2 Motivational Self System (Dörnyei 2009) by empirically testing a novel causal sequence—EI → grit → motivation → performance—thereby demonstrating how emotional regulation capacities can sustain perseverance and, in turn, reinforce motivational engagement. Secondly, at the methodological level, it adopts a large-scale SEM design (N = 801) with second-order and parcel-based modeling, using both questionnaire data and standardized CET-4 performance scores, which enhances measurement reliability and external validity. Thirdly, at the applied level, the study offers pedagogical insights for fostering emotionally intelligent, persistent, and motivated learners in high-stakes ESL contexts by suggesting classroom practices that integrate socio-emotional learning with goal-oriented instruction. These contributions provide a more comprehensive understanding of how emotional and motivational resources interact to shape successful second-language learning.

## 2. Literature Review

### 2.1. Control-Value Theory

Recent developments in educational psychology have emphasized that emotions are not merely by-products of learning but core determinants of cognitive and motivational functioning. The Control–Value Theory of Achievement Emotions (Pekrun 2006; Pekrun and Perry 2014) provides a comprehensive framework for understanding how emotional experiences influence learning outcomes. According to this theory, learners’ achievement emotions arise from their appraisals of control (the perceived ability to influence learning outcomes) and value (the perceived importance or intrinsic worth of the task). These emotions, in turn, shape motivation, engagement, and performance. Within this framework, emotional intelligence (EI) can be viewed as a critical personal resource that enhances both control and value appraisals: emotionally intelligent learners are more capable of regulating anxiety, sustaining enjoyment, and attributing personal significance to learning tasks. Consequently, EI may indirectly influence academic achievement through its effects on motivation and self-regulatory persistence, which aligns with the mediating roles of L2 grit and L2 motivation examined in the present study. By situating EI within the control–value perspective, this study contributes to a more integrated understanding of the emotional and motivational mechanisms underlying second-language learning.

### 2.2. Emotional Intelligence and Language Learning Performance

Emotional intelligence (EI), commonly defined as the ability to perceive, understand, regulate, and utilize emotions in oneself and others (Law et al. 2004; Mayer and Salovey 1997), has emerged as a key non-cognitive factor influencing educational outcomes. Research across disciplines shows that learners with higher EI are better at regulating anxiety, sustaining motivation, and adopting adaptive learning strategies that enhance academic performance (Babanoglu 2025; Gao et al. 2025). However, empirical findings on the EI–achievement relationship remain mixed. Yu and Tong (2024), in a longitudinal study of Chinese EFL learners, found that EI affected reading comprehension only indirectly through mediating cognitive variables such as word reading, vocabulary, and working memory, with the strength of mediation varying by text genre and socioeconomic background. In contrast, Peng and Li’s (2025) meta-analysis of 47 studies revealed a small-to-moderate but highly heterogeneous correlation between EI and language achievement (r = 0.24–0.41), noting that reported effects ranged from positive to even negative across contexts and depended on moderators such as learners’ educational level, target language, and skill domain. These contrasting results suggest that the contribution of EI to language performance is context-sensitive rather than uniform, and that EI may exert both direct and indirect influences depending on the nature of the learning task and population characteristics. This inconsistency highlights the need for further theory-driven investigations to clarify the mechanisms through which EI interacts with cognitive and motivational processes to shape L2 achievement.

**H1:**  *Emotional intelligence will positively predict the language learning performance mostly through indirect paths.*

### 2.3. Emotional Intelligence, L2 Grit, and Language Learning Performance

The concept of grit, originally introduced by Duckworth et al. (2007), refers to an individual’s sustained perseverance and passion for long-term goals. Within the field of second language acquisition (SLA), L2 grit captures learners’ persistence and consistency of interest toward language learning (Khajavy et al. 2025; Teimouri et al. 2022; Zhao and Wang 2023b). Numerous studies have linked grit with motivational engagement, willingness to communicate, and academic achievement (Zhao and Wang 2023a). For instance, Lake (2013) found that Japanese EFL learners with higher grit exhibited greater confidence and sustained effort, while Changlek and Palanukulwong (2015) observed similar patterns among Thai learners. In Chinese contexts, Wei et al. (2019) showed that grit positively predicted English course grades. Zhao et al. (2024) demonstrated that foreign language enjoyment (FLE) mediated the relationship between grit and online L2 Chinese achievement, highlighting the interplay between affective and motivational factors in second language learning. Similarly, Sun et al. (2024) found that perseverance of effort and consistency of interest jointly predicted young heritage learners’ speaking performance in Chinese through the serial mediation of motivational intensity and willingness to communicate, underscoring the motivational pathways through which grit facilitates L2 achievement.

However, some scholars have also questioned the structural and cultural generalizability of grit. Credé et al. (2017), in a meta-analytic review, argued that grit’s two dimensions—Perseverance of Effort (PE) and Consistency of Interest (CI)—are highly correlated with conscientiousness and that CI, in particular, shows weak or inconsistent predictive validity for academic success. They further suggested that grit may not be a unitary construct, but rather a repackaging of established personality traits. In collectivist settings, Datu et al. (2016) found that only PE significantly predicted academic engagement and well-being among Filipino students, whereas CI failed to load meaningfully on higher-order grit factors and was unrelated to achievement outcomes. Their cross-cultural analyses suggested that cultural values emphasizing social harmony and adaptability may render interest consistency less adaptive than effort perseverance, as students often adjust their goals to meet familial or social expectations rather than maintain fixed personal interests.

These critical perspectives imply that the relative importance of grit’s two dimensions may vary cross-culturally and that treating grit as a monolithic construct could obscure meaningful differences in how perseverance and consistency function across contexts. In line with Teimouri et al. (2022) and Sudina et al. (2021), the present study retains both PE and CI subdimensions to ensure comparability with prior L2 grit research while recognizing that PE may serve as the more salient predictor of motivational and performance outcomes in Chinese ESL learners. By incorporating both components, the study seeks not only to evaluate grit’s mediating role between emotional intelligence and achievement but also to contribute to the broader discussion about the cultural validity and dimensional specificity of grit in SLA.

**H2:**  *L2 grit will mediate the relationship between emotional intelligence and language learning performance.*

### 2.4. Emotional Intelligence, L2 Motivation, and Language Learning Performance

Motivation has long been regarded as a cornerstone of second language acquisition (SLA). Classic frameworks, such as Gardner’s (1985) socio-educational model and Dörnyei’s (2005) L2 motivational self-system, consistently demonstrate that motivated learners are more likely to initiate, sustain, and intensify their engagement with language tasks, leading to better achievement outcomes. Meta-analytic evidence further highlights motivation as one of the strongest predictors of language success across proficiency levels and learning contexts (Al-Hoorie 2018). Motivation functions not only as a direct driver of achievement but also as a mediator linking various cognitive and non-cognitive factors to performance (Fan and Wang 2025; Göksu and Louis 2025).

Emotional intelligence (EI) is expected to influence motivation through its effects on emotional regulation and the experience of achievement-related emotions. According to Pekrun’s (2006) Control-Value Theory, learners’ perceptions of control over learning and the value they assign to language learning are shaped by emotional experiences. High-EI learners are more capable of regulating negative emotions such as anxiety and frustration, while also amplifying positive emotions such as enjoyment and hope, which in turn sustain higher levels of motivation (MacIntyre and Gregersen 2012). For example, Coronado-Maldonado et al. (2025) found that university learners with higher EI demonstrated stronger intrinsic and extrinsic motivation, which mediated the effect of EI on academic performance. Similarly, Yu and Tong (2024) reported that EI indirectly enhanced Chinese EFL learners’ reading comprehension by increasing their engagement and motivational effort.

Empirical studies further indicate that EI facilitates motivational regulation, a key process in maintaining long-term language learning engagement. Gao et al. (2025) observed that EI not only predicted ESL learners’ achievement but also strengthened their willingness to communicate and invest sustained effort. In parallel, Peng and Li’s (2025) meta-analysis found that the EI–achievement relationship was significantly moderated by learners’ motivational variables. These findings underscore the view that EI is a crucial antecedent of motivation and that motivation itself plays a central role in translating EI into language achievement.

**H3:**  *L2 motivation will mediate the relationship between emotional intelligence and language learning performance.*

### 2.5. Emotional Intelligence, L2 Grit, L2 Motivation, and Language Learning Performance: A Chain Mediation Model

While both grit and motivation have been identified as key mediators linking emotional intelligence (EI) to achievement, recent theoretical advances suggest that these two constructs operate sequentially rather than independently. Grit, conceptualized as perseverance and sustained passion for long-term goals (Duckworth et al. 2007; Teimouri et al. 2022), reflects a relatively stable disposition that enables learners to persist despite setbacks. In contrast, motivation represents the dynamic, situation-specific drive that directs and energizes engagement in particular learning tasks (Gardner 1985; Dörnyei 2005). From a temporal perspective, grit provides the stamina to maintain effort over extended periods, thereby reinforcing motivational intensity once learners experience incremental progress or self-efficacy gains (Dörnyei and Ushioda 2011).

Integrating contemporary perspectives such as the L2 Motivational Self System (Dörnyei 2009) and the Expectancy–Value Framework (Wigfield and Eccles 2000), grit can be viewed as the dispositional foundation that sustains learners’ expectancy of success and value appraisals over time. Emotionally intelligent learners—capable of regulating anxiety and maintaining optimism—are more likely to persevere (grit) in the face of difficulty, which in turn stabilizes and strengthens self-determined motivation. In this sense, grit precedes motivation, functioning as the self-regulatory mechanism that converts emotional resources into goal-directed persistence and renewed motivational energy.

This interpretation aligns with evidence from Self-Determination Theory (SDT), which emphasizes that the satisfaction of basic psychological needs fuels resilience and autonomous motivation (Ryan and Deci 2020). Empirical studies in educational psychology support this chain: Trigueros and García-Mas (2025) demonstrated that novelty and self-determination predicted resilience and grit, which subsequently enhanced motivation and engagement; Trigueros et al. (2025) similarly found that peer support and need satisfaction fostered grit and intrinsic motivation among adolescents, highlighting grit’s mediating role between need satisfaction and motivational outcomes. In the language domain, Fernandez-Ortega et al. (2024) reported that frustration of psychological needs heightened negative emotions (e.g., anxiety, embarrassment), which undermined motivation and communicative intention in English, reinforcing the critical link between emotional regulation, grit-like perseverance, and motivational engagement.

These findings suggest that emotionally intelligent learners are more capable of sustaining perseverance (grit), which in turn consolidates self-determined motivation—both essential precursors of successful language learning. This sequential mechanism bridges emotional regulation, long-term effort, and motivational energy, offering a coherent theoretical account of how EI translates into achievement in second-language contexts.

**H4:**  *Emotional intelligence will predict language learning performance through the sequential mediation of L2 grit and L2 motivation.*

**Figure 1:**
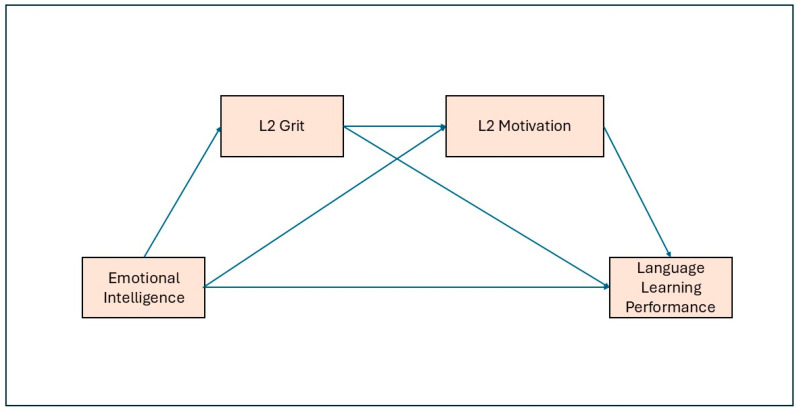
Conceptual model of the hypothesized relationships among emotional intelligence, L2 grit, L2 motivation, and language learning performance.

Building on prior research linking noncognitive resources with language learning outcomes, the present study proposes a conceptual framework (see Figure 1) in which emotional intelligence (EI) exerts both direct and indirect effects on performance. As illustrated in Figure 1, EI is expected to predict L2 grit (i.e., perseverance of effort and consistency of interest) and L2 motivation (i.e., motivated learning behaviors), which in turn serve as mediating mechanisms. The model also hypothesizes a sequential pathway, whereby learners high in EI demonstrate greater grit, which subsequently enhances motivation and ultimately leads to improved language learning performance. This integrative framework reflects recent calls to consider socio-emotional, motivational, and performance-related constructs within a unified model of second language acquisition (e.g., Dörnyei 2005; MacCann et al. 2020; Teimouri et al. 2019).

## 3. Methods

### 3.1. Participants

The participants in this study were Chinese university students enrolled in English-as-a-Second Language (ESL) courses at seven comprehensive universities located in eastern China. The institutions were selected through convenience sampling, based on their comparable curriculum structures and accessibility for data collection. Within each university, instructors distributed the online questionnaire to all students enrolled in intermediate or advanced English courses. Participation was entirely voluntary, and students were informed that their responses would remain anonymous and would not affect their academic standing.

Following the recommendations of Jackson (2003) regarding the sample-size-to-free-parameters (N:q) ratio, we determined the required sample size for our model. Jackson (2003) provides empirical support for this guideline, suggesting it is a more reliable heuristic than rules based on the number of observed variables, as it directly accounts for model complexity. Our proposed model requires an estimation of 31 free parameters (q = 31). Applying the commonly recommended 10:1 ratio (Jackson 2003), our target sample size was established at 310 participants (31 parameters × 10). This sample size is considered sufficient to ensure stable parameter estimates, accurate standard errors, and adequate statistical power.

A total of 871 students completed the survey, of whom 472 were male and 399 were female, with an average age of 21.2 years (SD = 1.7). The inclusion criteria required that participants (a) had studied English for at least six consecutive years, (b) had taken the national English proficiency examination for college students in China, CET-4 (c) identified Chinese as their first language. All participants were bilingual (Chinese English), and none reported multilingual proficiency beyond English.

Of the total responses, 70 were excluded due to missing or invalid data, yielding a final sample of 801 students (432 males, 369 females; M_age = 21.1, SD = 1.7). The average response rate across universities was about 80%.

### 3.2. Instruments

All instruments were administered in their validated Chinese versions. To guarantee the precision of these translations, the questionnaire underwent a thorough review by two professional English translation students and an authoritative expert in applied linguistics.

#### 3.2.1. Emotional Intelligence (EI)

EI was measured using the Self-Report Emotional Intelligence Scale (WLEIS) (Wong and Law 2002), which has been widely applied in Chinese contexts and demonstrates robust psychometric properties. The scale consists of 16 items across four dimensions: self-emotion appraisal (SEA), others’ emotion appraisal (OEA), use of emotion (UOE), and regulation of emotion (ROE). Responses were recorded on a 5-point Likert scale (1 = strongly disagree, 5 = strongly agree). Previous studies with Chinese EFL learners have reported Cronbach’s α values above 0.85 (Gao et al. 2025). Prior to the main survey, a pilot test was conducted with 78 Chinese university students to examine the psychometric equivalence between the Chinese and the original English versions. Exploratory factor analysis (EFA) confirmed a four-factor structure consistent with the original WLEIS, and all item loadings exceeded 0.65. The subscale reliabilities and overall Cronbach’s α were above 0.70, indicating satisfactory internal consistency and structural equivalence across versions.

#### 3.2.2. L2 Grit

L2 grit was measured using the L2-Grit Scale (Teimouri et al. 2022), which was designed specifically for second language learning contexts and has been validated in multiple EFL/ESL settings (Sudina et al. 2021). The scale consists of nine items tapping two dimensions: Perseverance of Effort (five items; e.g., “I put much time and effort into improving my English language weaknesses”) and Consistency of Interest (four items; e.g., “My interests in learning English change from year to year”). Responses were rated on a 5-point Likert scale ranging from 1 to 5. Consistent with prior research, negatively worded items in the Consistency of Interest subscale were reverse-coded before analysis (Sudina et al. 2021). Previous validation studies reported acceptable internal consistency (α > 0.80) and confirmed a two-factor structure via exploratory and confirmatory factor analyses (Teimouri et al. 2022; Sudina et al. 2021). A pilot study involving 78 Chinese university students was conducted to assess the psychometric equivalence of the adapted version. Exploratory factor analysis (EFA) supported a two-factor structure consistent with the original model, with all item loadings exceeding 0.60. The Cronbach’s α values for the PE and CI subscales were 0.82 and 0.78, respectively, and the overall reliability was above 0.75, demonstrating satisfactory internal consistency and factorial validity in the Chinese context.

#### 3.2.3. L2 Motivation

L2 motivation was assessed using the Motivated Learning Behavior subscale from Papi and Teimouri’s (2014) validated questionnaire on language learner motivational types. This subscale specifically captures learners’ intended effort and persistence in studying English, reflecting their behavioral engagement with language learning. It consists of six items (e.g., “I would like to spend lots of time studying English”; “I am prepared to expend a lot of effort in learning English”; “If an English course was offered in the future, I would like to take it”). Responses were provided on a 5-point Likert scale ranging from 1 (strongly disagree) to 5 (strongly agree). The original study reported satisfactory internal consistency (α = 0.80), and subsequent applications in SLA research have confirmed the reliability of this subscale across diverse contexts (α = 0.90) (Wu 2024). A pilot study with 78 Chinese university students was conducted to verify the psychometric properties of the adapted version. Exploratory factor analysis (EFA) revealed a single-factor structure consistent with the original instrument, with all item loadings exceeding 0.65. The Cronbach’s α for the subscale was 0.75, indicating strong internal consistency and satisfactory factorial validity for the Chinese version of the scale.

### 3.3. Language Learning Performance

Language achievement was operationalized using participants’ most recent self-reported College English Test Band 4 (CET-4) scores, a standardized nationwide English proficiency exam administered by the Ministry of Education in China. CET-4 is widely recognized as a reliable and valid indicator of university students’ English proficiency and has been frequently employed as a criterion variable in second language acquisition (SLA) research (Yu and Tong 2024). In this study, participants reported their most recent CET-4 scores obtained within six months prior to data collection. The use of self-reported scores was necessary due to the anonymous nature of the survey, which did not collect identifying information such as names or student IDs. Prior evidence indicates that self-reported standardized test scores are generally reliable when anonymity is ensured and no incentives for misreporting exist (Cassidy 2012; Kuncel et al. 2005).

### 3.4. Procedure

The study was conducted in the spring semester of 2024. After obtaining ethical approval from the university’s Institutional Review Board, participants were recruited during regular English classes. Students were first provided with detailed instructions and assured of anonymity and confidentiality. Completion of the questionnaire required approximately 20 min.

### 3.5. Data Analysis

Data were analyzed using R and Lavaan. First, descriptive statistics and reliability coefficients (Cronbach’s α and McDonald’s ω) were calculated for all scales. Next, Confirmatory Factor Analysis (CFA) was conducted to validate the measurement models of EI, L2 grit, and L2 motivation. Model fit was evaluated using χ^2^/df, CFI, TLI, RMSEA, and SRMR indices.

To enhance model parsimony and reduce random measurement error, we adopted the item parceling approach when testing the hypothesized chain mediation model. Specifically, all items within each subscale were averaged to create a single parcel representing that dimension (e.g., the four items of Self-Emotion Appraisal [SEA] were aggregated into one SEA parcel). In this way, the measurement model included four parcels for Emotional Intelligence (SEA, OEA, UOE, ROE), two parcels for L2 Grit (Perseverance of Effort [PE], Consistency of Interest [CI]), and no parcel for L2 Motivation (Motivated Learning Behavior [MOT]) since this latent construct is identical with its subscale. The latent construct “Performance” was directly represented by the observed test score. This strategy is consistent with recommendations in SEM literature that parceling can yield more stable solutions and improve distributional properties when subscales are theoretically unidimensional (Little et al. 2002).

To test the hypothesized mediation model, structural equation modeling (SEM) with maximum likelihood estimation was performed. Both direct and indirect effects were estimated, and the significance of mediation was assessed using bias-corrected bootstrap confidence intervals (5000 resamples). The hypothesized chain mediation (EI → L2 grit → L2 motivation → performance) was compared against alternative models (e.g., single mediation models) to evaluate explanatory power.

## 4. Results

### 4.1. Descriptive Statistics

Table 1 presents the descriptive statistics for the four latent constructs: Emotional Intelligence (EI), L2 Grit, L2 Motivation (MOT), and Performance. The mean scores for EI (M = 3.01, SD = 0.87), GRIT (M = 3.10, SD = 0.95), and MOT (M = 3.04, SD = 1.07) indicate that learners reported moderate levels of emotional competence, perseverance, and motivated learning behavior. Performance scores followed the expected distribution of standardized test outcomes (M = 500, SD = 80). Across all constructs, skewness and kurtosis values suggest no substantial deviation from normality and support the suitability of these variables for further structural analyses.

Table 2 displays the descriptive statistics for the subscales. The four EI subscales showed comparable means, with Self-Emotion Appraisal (SEA: M = 3.10, SD = 1.10), Others’ Emotion Appraisal (OEA: M = 2.95, SD = 1.09), Use of Emotion (UOE: M = 2.91, SD = 1.10), and Regulation of Emotion (ROE: M = 3.05, SD = 1.11) all clustering around the scale midpoint. For GRIT, Perseverance of Effort (PE) showed a slightly higher mean (M = 3.15, SD = 1.06) than Consistency of Interest (CI: M = 3.05, SD = 1.10). The single subscale of L2 Motivation, Motivated Learning Behavior (MOT), had a mean of 3.04 (SD = 1.07), nearly identical to the latent-level average. The distributional indices for all subscales were within acceptable ranges (Skew = −0.03 to 0.02; Kurtosis = −1.06 to −0.96), indicating approximate normality.

### 4.2. Reliability and Validity

A second-order confirmatory factor analysis (CFA) was conducted on the collected data to evaluate the measurement model. The model demonstrated a good fit to the data, χ^2^(425) = 1442.87, *p* < .001, CFI = 0.98, TLI = 0.98, RMSEA = 0.06, 90% CI [0.05, 0.06], SRMR = 0.05. Based on this well-fitting measurement model, we subsequently computed the reliability and validity indices (CR and AVE) for each latent construct. The factor loadings of the second-order CFA are presented in Appendix A.

Table 3 reports the reliability and validity indices at both the latent and subscale levels. At the latent level, Emotional Intelligence (EI) and L2 Grit (GRIT) demonstrated adequate convergent validity, with average variance extracted (AVE) values of 0.64 and 0.69, respectively. The composite reliability (CR) values for EI (0.88) and GRIT (0.82) were above the recommended threshold of 0.70, and Cronbach’s alpha values indicated acceptable internal consistency (α = 0.80 and 0.79, respectively). L2 Motivation (MOT), assessed as a latent variable from six indicators, showed strong reliability (α = 0.86, CR = 0.88) and AVE at 0.69.

At the subscale level, all measures exhibited satisfactory psychometric properties. The four EI subscales—Self-Emotion Appraisal (SEA: AVE = 0.53, CR = 0.81, α = 0.78), Others’ Emotion Appraisal (OEA: AVE = 0.51, CR = 0.80, α = 0.76), Use of Emotion (UOE: AVE = 0.53, CR = 0.82, α = 0.80), and Regulation of Emotion (ROE: AVE = 0.55, CR = 0.83, α = 0.80)—all met the conventional thresholds for convergent validity and reliability. The two GRIT subscales, Perseverance of Effort (PE: AVE = 0.51, CR = 0.83, α = 0.81) and Consistency of Interest (CI: AVE = 0.52, CR = 0.81, α = 0.77), likewise demonstrated solid reliability and construct validity. The MLB subscale of MOT also achieved reliability and construct validity (AVE = 0.55, CR = 0.88, α = 0.86), reinforcing its robustness as a measure of motivated learning behavior.

### 4.3. Correlation

The correlations among the four latent constructs are shown in Table 4. Emotional Intelligence (EI) was significantly and positively correlated with L2 Grit (*r* = 0.43, *p* < .001), L2 Motivation (*r* = 0.55, *p* < .001), and Performance (*r* = 0.66, *p* < .001). Similarly, GRIT was strongly associated with MOT (*r* = 0.57, *p* < .001) and with Performance (*r* = 0.63, *p* < .001). Among all constructs, the strongest association was observed between MOT and Performance (*r* = 0.81, *p* < .001), indicating that motivated learning behavior was the most direct predictor of achievement outcomes. Although the correlation between L2 motivation and performance was relatively high (r = 0.81), this association is theoretically expected given that motivation directly drives language learning effort, which in turn determines standardized test outcomes. Moreover, performance was measured through an external standardized exam (CET-4), reducing the likelihood of common-method bias.

As is shown in Table 5, within EI, the four subscales—Self-Emotion Appraisal (SEA), Others’ Emotion Appraisal (OEA), Use of Emotion (UOE), and Regulation of Emotion (ROE)—were moderately intercorrelated (*r*s = 0.47–0.52, *p*s < 0.001), supporting their coherence as components of EI. The two GRIT subscales, Perseverance of Effort (PE) and Consistency of Interest (CI), were strongly correlated (*r* = 0.53, *p* < .001). Across constructs, PE and CI were significantly associated with all EI subscales (*r*s = 0.25–0.36, *p*s < 0.001) and with Motivated Learning Behavior (MOT; *r* = 0.57 and 0.43, respectively, *p*s < 0.001). MLB was further correlated with each EI subscale (*r*s = 0.40–0.48, *p*s < 0.001), suggesting that emotional competence and perseverance are closely tied to motivational engagement in L2 learning.

### 4.4. SEM Results

#### 4.4.1. Model Fit Statistics

We first tested the model according to the conceptual framework, but the initial model exhibited suboptimal fit (χ^2^(60) = 498.47, *p* < .001, CFI = 0.91, TLI = 0.89, RMSEA = 0.10, 90% CI [0.09, 0.10], SRMR = 0.06). We then examined the modification indices and found high residual correlations between the first and second items of the MOT construct. A review of the questionnaire confirmed that these items were conceptually related, justifying the addition of corresponding covariance parameters between their residuals. After including these two residual covariances, the model achieved a satisfactory overall fit (see Table 6).

The hypothesized chain mediation model demonstrated a satisfactory fit to the data (Table 6). The chi-square test was significant, χ^2^(59) = 302.11, *p* < .001, which is expected with large sample sizes. Other indices indicated an acceptable to good model fit: CFI = 0.953 and TLI = 0.937, both exceeding the conventional 0.90 criterion and approaching the more stringent 0.95 threshold. The RMSEA was 0.072 (90% CI [0.064, 0.080]), falling within the acceptable range below 0.08, and the SRMR value of 0.048 was well below the recommended 0.08 cutoff. Collectively, these indices indicate that the revised chain mediation model adequately represents the observed data and achieves a substantial improvement over the initial model.

#### 4.4.2. Standardized Path Coefficients

The structural model revealed several significant direct effects (see Table 7). Emotional Intelligence (EI) strongly predicted GRIT (β = 0.574, SE = 0.036, z = 15.800, *p* < .001, 95% CI [0.503, 0.645]). Both EI (β = 0.426, SE = 0.043, z = 10.016, *p* < .001, 95% CI [0.343, 0.510]) and GRIT (β = 0.515, SE = 0.043, z = 11.919, *p* < .001, 95% CI [0.430, 0.599]) significantly predicted Motivation (MOT), indicating that EI not only directly enhances motivational engagement but also bolsters MOT through increased perseverance. For academic outcomes, MOT emerged as the strongest predictor of Performance (β = 0.635, SE = 0.049, z = 12.916, *p* < .001, 95% CI [0.539, 0.732]), with additional—though smaller—direct effects from EI (β = 0.185, SE = 0.034, z = 5.432, *p* < .001, 95% CI [0.118, 0.251]) and GRIT (β = 0.165, SE = 0.041, z = 4.026, *p* < .001, 95% CI [0.085, 0.246]). Overall, these findings suggest that EI contributes to Performance both directly and indirectly via GRIT and MOT, consistent with the proposed chain pathway EI → GRIT → MOT → Performance.

### 4.5. Indirect Effects

Table 8 presents the bootstrap results for the standardized indirect effects of Emotional Intelligence (EI) on Performance. EI exerted a significant indirect effect on Performance through GRIT (β = 0.095, *p* < .001) and through MOT (β = 0.271, *p* < .001). In addition, the hypothesized chain mediation pathway (EI → GRIT → MOT → Performance) was also significant (β = 0.188, *p* < .001). The total indirect effect of EI on Performance was substantial (β = 0.553, *p* < .001), indicating that the majority of the influence of EI on achievement operated through GRIT and MOT. The overall total effect of EI on Performance was strong (β = 0.738, *p* < .001), underscoring the central role of EI in predicting language learning outcomes primarily via motivational and perseverance-related mechanisms.

In terms of explained variance (see Table 9), the model accounted for a meaningful proportion across mediators and the outcome. Specifically, GRIT showed an *R*^2^ of 0.329, MOT had an *R*^2^ of 0.549, and Performance reached an *R*^2^ of 0.787. These findings indicate that EI and its mediating pathways explained approximately one-third of the variance in grit, over half of the variance in motivation, and nearly 80% of the variance in learners’ performance. Such high levels of explained variance demonstrate the strong explanatory power of the proposed chain mediation model. To assess potential multicollinearity among latent predictors, we computed variance inflation factors (VIFs) based on the latent correlation matrix derived from the SEM. For the regression predicting MOT (EI + GRIT), VIFs were 1.49 for both EI and GRIT (Tolerance = 0.67), indicating negligible collinearity. For Performance (EI + GRIT + MOT), VIFs were 2.09, 2.37, and 3.31 for EI, GRIT, and MOT, respectively (Tolerances = 0.48–0.30). All values were well below the conventional thresholds (VIF < 5, Tolerance > 0.20), suggesting that multicollinearity did not bias the structural estimates.

**Figure 2:**
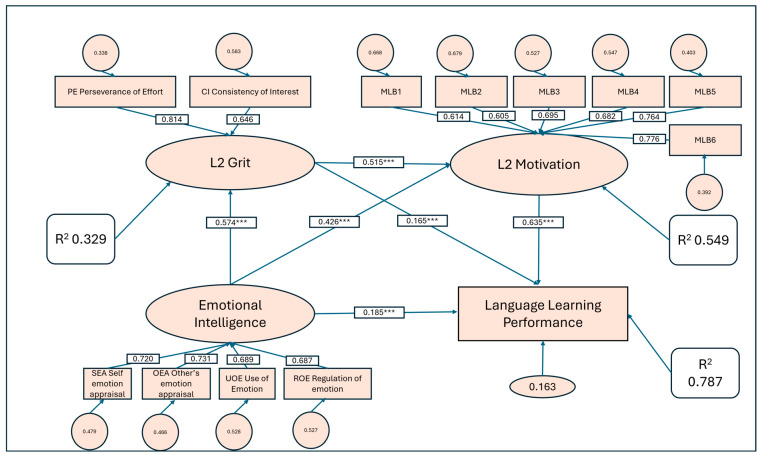
Structural equation model illustrating the relationships among emotional intelligence, L2 grit, L2 motivation, and language learning performance, with standardized path coefficients, loadings, residuals and *R*^2^ values. *** *p* < 0.001.

All standardized coefficients, indirect effects, and explained variances (*R*^2^) are summarized visually in Figure 2. This structural model provides a comprehensive overview of the tested mediation pathways.

## 5. Discussion

The hypothesized model revealed several significant associations among emotional intelligence (EI), grit, motivation, and performance (Table 7). EI was positively associated with grit (β = 0.574, SE = 0.036, z = 15.800, *p* < .001, 95% CI [0.503, 0.645]) and motivation (β = 0.426, SE = 0.043, z = 10.016, *p* < .001, 95% CI [0.343, 0.510]). Grit was also strongly correlated with motivation (β = 0.515, SE = 0.043, z = 11.919, *p* < .001, 95% CI [0.430, 0.599]). In relation to performance, motivation (β = 0.635, SE = 0.049, z = 12.916, *p* < .001, 95% CI [0.539, 0.732]), EI (β = 0.185, SE = 0.034, z = 5.432, *p* < .001, 95% CI [0.118, 0.251]), and grit (β = 0.165, SE = 0.041, z = 4.026, *p* < .001, 95% CI [0.085, 0.246]) all showed significant positive relations with the outcome.

Bootstrap analysis further revealed robust indirect associations (Table 8). EI was indirectly related to performance through grit (β = 0.095, SE = 0.025, z = 3.781, *p* < .001, 95% CI [0.048, 0.149]), through motivation (β = 0.271, SE = 0.039, z = 7.008, *p* < .001, 95% CI [0.199, 0.350]), and via the sequential path EI–grit–motivation–performance (β = 0.188, SE = 0.026, z = 7.326, *p* < .001, 95% CI [0.140, 0.241]). The total indirect association was β = 0.553 (SE = 0.039, z = 14.048, *p* < .001, 95% CI [0.476, 0.628]), and the total association between EI and performance reached β = 0.738 (SE = 0.033, z = 22.423, *p* < .001, 95% CI [0.669, 0.805]). Collectively, these findings indicate that EI, grit, and motivation were closely interrelated, with both direct and indirect links to performance, underscoring the strong interconnectedness among the four constructs.

The findings of this study are consistent with Hypothesis 1, showing that emotional intelligence (EI) is positively associated with language learning performance (β = 0.185, *p* < .001). This result indicates that learners with higher levels of EI tend to achieve better academic outcomes in English as a second language. The direct effect of EI on performance aligns with previous research emphasizing the crucial role of emotional competencies in academic success. For instance, MacCann et al. (2020) found that EI predicted achievement above and beyond cognitive ability and personality, while Li and Xu (2019) reported that students with stronger emotion regulation and appraisal skills were better able to cope with language learning anxiety and sustain effort, thereby achieving higher levels of performance. Similar conclusions were drawn by Petrides et al. (2004) and Perera and DiGiacomo (2013), who highlighted EI as a predictor of both academic engagement and achievement. At the same time, the present findings differ from some earlier studies where EI’s influence on performance was found to be mostly indirect, mediated by motivational or affective variables (Dewaele 2019). One explanation for this discrepancy may be methodological. Whereas prior studies often modeled EI’s effects indirectly through mediators such as motivation, the present study employed a parcel-based SEM approach, which reduces measurement error and allows for more reliable estimation of direct paths (Little et al. 2002). This approach may have revealed a stronger direct effect of EI on performance than previously observed. These results suggest that EI is not only a distal resource that operates through mediating mechanisms such as grit and motivation but also a proximal predictor of achievement. Learners who can effectively perceive, regulate, and use emotions may directly channel these capacities into improved focus, resilience, and learning strategies, thereby enhancing their language learning performance (Mayer and Salovey 1997; Pekrun 2006).

The results are consistent with Hypothesis 2, indicating that L2 grit significantly mediated the relationship between EI and language learning performance. From a correlational standpoint, emotional intelligence (EI) showed a strong positive association with grit (β = 0.574, SE = 0.036, z = 15.800, *p* < .001, 95% CI [0.503, 0.645]), and grit was in turn positively related to performance (β = 0.165, SE = 0.041, z = 4.026, *p* < .001, 95% CI [0.085, 0.246]). Bootstrap results indicated a significant indirect association between EI and performance through grit (β = 0.095, SE = 0.025, z = 3.781, *p* < .001, 95% CI [0.048, 0.149]), suggesting that individuals with higher EI also tended to report higher grit levels, which were in turn associated with stronger performance outcomes, confirming the mediating role of grit. This finding is consistent with prior studies emphasizing the role of grit in academic achievement and its links to socio-emotional resources. For instance, Duckworth et al. (2007) originally conceptualized grit as perseverance and passion for long-term goals, and subsequent work has shown its predictive validity for academic success across diverse contexts (Credé et al. 2017). Within the domain of language learning, Teimouri et al. (2019) and Wei et al. (2019) demonstrated that L2 grit predicted language proficiency and persistence in learning, while Sudina and Plonsky (2021) noted that learners with stronger emotional and motivational regulation tend to maintain higher levels of perseverance. The present study extends this body of work by providing evidence that EI functions as an antecedent of L2 grit, highlighting the pathway through which emotional competencies facilitate sustained effort and resilience in second-language learning. In line with socio-emotional theories of learning (Mayer and Salovey 1997; Pekrun 2006), learners who can effectively perceive, regulate, and utilize their emotions are more likely to persevere despite difficulties, which in turn enhances academic outcomes.

The findings are also consistent with Hypothesis 3, showing that L2 motivation mediated the relationship between EI and performance. From a correlational perspective, emotional intelligence (EI) was positively associated with motivation (β = 0.426, SE = 0.043, z = 10.016, *p* < .001, 95% CI [0.343, 0.510]), and motivation was in turn strongly related to performance (β = 0.635, SE = 0.049, z = 12.916, *p* < .001, 95% CI [0.539, 0.732]). Bootstrap analysis indicated a significant indirect association and mediation between EI and performance through motivation (β = 0.271, SE = 0.039, z = 7.008, *p* < .001, 95% CI [0.199, 0.350]). These results suggest that individuals with higher emotional intelligence also tended to report greater motivation, which was closely linked with higher performance outcomes. These results are consistent with previous studies demonstrating that emotionally intelligent learners tend to sustain higher levels of motivation in language learning (Dewaele and Li 2020; Gardner 2010; Papi and Khajavy 2021). Learners who can effectively regulate emotions are better able to maintain interest, exert effort, and remain engaged in learning tasks, which translates into stronger performance (Dörnyei and Ushioda 2011; Pekrun 2006). The findings also align with expectancy-value theory (Eccles and Wigfield 2002) and self-determination theory (Ryan and Deci 2000), both of which posit that motivational engagement serves as a key mechanism through which personal traits influence academic outcomes.

The results are consistent with Hypothesis 4, which proposed a sequential mediation pathway from EI to performance through L2 grit and L2 motivation. From a correlational standpoint, emotional intelligence (EI), grit, motivation, and performance were strongly interconnected through a sequential pattern. EI was positively associated with grit (β = 0.574, *p* < .001), grit was positively related to motivation (β = 0.515, *p* < .001), and motivation, in turn, was positively linked with performance (β = 0.635, *p* < .001). The sequential indirect association following the chain EI → grit → motivation → performance was statistically significant (β = 0.188, SE = 0.026, z = 7.326, *p* < .001, 95% CI [0.140, 0.241]). This pattern indicates that higher emotional intelligence tended to co-occur with greater grit and motivation, which together were associated with higher performance, highlighting a coherent chain of associations among the constructs. This finding is consistent with theoretical frameworks positing that socio-emotional competencies affect achievement indirectly through motivational processes (Pekrun 2006). Prior research has shown that learners high in EI are more likely to persist in the face of difficulties (Mayer and Salovey 1997; MacCann et al. 2020), which reflects higher levels of grit (Duckworth et al. 2007; Teimouri et al. 2022). In turn, grit has been found to sustain motivation over time by supporting learners’ ability to maintain interest and invest effort in language learning (Wei et al. 2019; Sudina and Plonsky 2021). Motivation itself has long been identified as a proximal driver of achievement in second language acquisition (Dörnyei 2005; Papi and Khajavy 2021), serving as the immediate mechanism through which persistence and engagement are translated into performance gains. Compared with earlier studies that typically examined either the EI–motivation–achievement pathway (Dewaele and Li 2020; MacCann et al. 2020) or the grit–achievement link in isolation (Teimouri et al. 2022), the present study demonstrates the added value of considering grit and motivation together in a sequential model. By integrating these two mediators, the findings highlight how EI not only initiates perseverance but also channels this perseverance into sustained motivational energy, which then enhances academic performance. The strong explanatory power of the sequential mediation model (*R*^2^ = 0.787 for performance) further underscores the robustness of this pathway.

From a pedagogical perspective, the findings suggest that interventions targeting emotional intelligence may indirectly improve learners’ perseverance, motivation, and, ultimately, language achievement. Teachers can integrate socio-emotional learning activities into the curriculum, such as reflective tasks, emotion regulation strategies, and collaborative exercises that promote empathy and interpersonal awareness. Furthermore, supporting students in setting long-term learning goals and reinforcing consistent effort can strengthen grit, which, in turn, fuels motivation. Institutional policies that recognize the role of socio-emotional development in language learning could also enhance student engagement and persistence.

### 5.1. Implications

From a theoretical perspective, this study extends the Control–Value Theory (Pekrun 2006) by clarifying how emotional intelligence (EI) influences language learning performance through L2 grit and motivation. The findings indicate that EI provides learners with the emotional regulation and perceived control necessary to sustain long-term learning efforts and value their academic goals, which, in turn, facilitates motivational engagement and perseverance. This integrated emotional–motivational model highlights the interconnected nature of affective and volitional factors in shaping second language achievement and offers an expanded framework for understanding individual differences in L2 learning.

From a pedagogical perspective, the results provide actionable guidance for language educators seeking to foster socio-emotional and motivational growth among learners. Firstly, teachers should actively cultivate learners’ emotional intelligence by integrating socio-emotional learning (SEL) into daily instruction. This may include reflective journaling on emotional experiences in language learning, role-play and storytelling tasks that develop empathy and emotional awareness, and guided discussions on how to manage frustration and anxiety during language use. Secondly, to strengthen L2 grit, educators should help students set specific and attainable learning goals, monitor their own progress, and celebrate incremental achievements to reinforce self-efficacy. Incorporating materials and activities that connect with learners’ personal interests, lived experiences, and cultural backgrounds can also sustain engagement and long-term commitment to language learning. Thirdly, enhancing L2 motivation requires creating a supportive and autonomy-oriented classroom environment. Teachers can offer meaningful choices in learning activities, connect lessons to real-world communicative purposes, and provide opportunities for goal reflection to strengthen intrinsic motivation. Finally, given the variability in students’ emotional intelligence, grit, and motivation, teachers should adopt differentiated instruction and personalized feedback strategies to address individual differences effectively.

At the institutional level, curriculum designers and administrators should recognize socio-emotional competencies as foundational to successful L2 learning. Embedding emotion regulation, perseverance, and motivational training into teacher development programs and assessment frameworks can cultivate classrooms where learners are not only linguistically competent but also emotionally resilient and self-motivated.

In addition, the present findings point to promising but as yet untested avenues for intervention. Future research could design pilot EI-based training programs, such as emotional regulation workshops, mindfulness and empathy sessions, or self-reflective emotional awareness activities, followed by systematic evaluation of changes in learners’ motivation and performance. These interventions could be tested through experimental or quasi-experimental designs using pre–post assessments and control groups to determine whether improvements in emotional intelligence correspond to increases in motivation and achievement. Researchers should also account for potential covariates, including learners’ cognitive ability, prior proficiency, and institutional support, to isolate the specific effects of socio-emotional training. Such pilot programs would offer empirical evidence for the causal direction of the observed associations and guide the development of sustainable, evidence-based emotional intelligence interventions in language education.

### 5.2. Limitations and Future Directions

Despite its contributions, this study has several limitations that warrant caution. Firstly, the use of self-report questionnaires for EI, grit, and motivation may introduce common method bias and social desirability effects. Although structural modeling helped address measurement error, future research could benefit from incorporating multi-method assessments, such as teacher ratings or behavioral indicators. Secondly, the cross-sectional nature of this study limits the ability to draw causal conclusions regarding the relationships among emotional intelligence, grit, motivation, and performance. Although the observed associations are consistent with the hypothesized sequential pattern, the temporal order of these variables cannot be verified within a single time point. Thirdly, performance was measured using a standardized test, which may not fully capture learners’ communicative competence or real-world language use. Fourthly, although the present study, like many other studies (Li et al. 2021; Sudina et al. 2021; Sudina and Plonsky 2021), focused on emotional and motivational predictors, future research should integrate cognitive ability measures or contextual support measures to more comprehensively capture the control–value dynamics underlying language learning outcomes. Cognitive abilities such as working memory, attentional control, and reasoning capacity form the cognitive foundation upon which emotional and motivational processes operate. Fifthly, while item parceling was adopted to enhance model stability and reduce random measurement error, it may also obscure within-construct variability and lead to overestimation of reliability or model fit. Future research should re-estimate models using item-level indicators or conduct sensitivity analyses to assess the robustness of parcel-based results. Finally, the sample was drawn from a single cultural and educational context in China, which may constrain the generalizability of the findings to other L2 learning populations.

Building on these limitations, future research could explore several promising directions. Future studies should employ longitudinal designs to examine how these constructs evolve and influence one another over time, or experimental/intervention-based approaches to establish directional effects more rigorously. Intervention studies could test whether explicit training in emotional intelligence or perseverance strategies leads to measurable gains in motivation and performance. Expanding the scope of performance measures to include cognitive abilities, communicative competence, classroom participation, or teacher assessments may provide a more comprehensive understanding of outcomes. Furthermore, cross-cultural comparisons could reveal whether the observed mediation mechanisms hold across diverse educational systems and cultural contexts. Future research could also further explore potential gender differences in the emotional–motivational mechanisms underlying language learning. Given prior evidence that females often demonstrate higher levels of emotional intelligence and empathy (Cabello et al. 2016), a multi-group structural equation modeling (SEM) approach could be employed to compare whether the structural relations among EI, grit, motivation, and performance differ across male and female learners. Such analysis would provide valuable insights into gender-specific pathways of emotional and motivational functioning in second language learning. Finally, integrating additional socio-emotional constructs—such as foreign language enjoyment, anxiety, or resilience—could enrich theoretical models and offer a more complete picture of the emotional-motivational landscape in second language acquisition.

## 6. Conclusions

The present study examined how emotional intelligence (EI) predicts language learning performance through the sequential mediation of L2 grit and motivation. The findings confirmed that EI has both direct effects on performance and indirect effects via grit and motivation. Specifically, EI significantly predicted grit and motivation, which in turn contributed to higher levels of achievement. The sequential mediation pathway (EI → grit → motivation → performance) was also significant, highlighting the multi-step mechanisms through which emotional competencies shape learning outcomes. The model demonstrated strong explanatory power, accounting for 33% of the variance in grit, 55% in motivation, and nearly 80% in performance. These results underscore the central role of EI as both a proximal driver and a distal enabler of second language learning success. By integrating emotional and motivational perspectives, this study contributes to a more comprehensive understanding of how learners’ emotional competencies are translated into academic outcomes.

## Figures and Tables

**Table 1:** Descriptive Statistics for Latent Constructs.

Construct	N	M	SD	Min	Max	Skew	Kurt
EI	801	3.01	0.87	1	4.94	−0.03	−0.74
GRIT	801	3.10	0.95	1	5.00	−0.01	−0.85
MOT	801	3.04	1.07	1	5.00	−0.02	−0.99
Performance	801	500	80.00	227	710.00	−0.11	−0.11

**Table 2:** Descriptive Statistics for Subscales.

Subscale	N	M	SD	Min	Max	Skew	Kurt
SEA	801	3.10	1.10	1	5	0.00	−1.03
OEA	801	2.95	1.09	1	5	0.02	−0.96
UOE	801	2.91	1.10	1	5	0.00	−0.97
ROE	801	3.05	1.11	1	5	0.02	−0.96
PE	801	3.15	1.06	1	5	0.00	−0.97
CI	801	3.05	1.10	1	5	−0.03	−1.06
MOT	801	3.04	1.07	1	5	−0.02	−0.99

Note. Subscale scores are the mean of their items. Abbreviations: EI = Emotional Intelligence; SEA = Self-Emotion Appraisal; OEA = Others’ Emotion Appraisal; UOE = Use of Emotion; ROE = Regulation of Emotion; GRIT = L2 Grit; PE = Perseverance of Effort; CI = Consistency of Interest; MOT = L2 Motivation.

**Table 3:** Reliability and Validity.

Level	Construct	k (Indicators)	AVE	CR	Alpha
Latent	EI	4	0.64	0.88	0.80
Latent	GRIT	2	0.69	0.82	0.79
Latent	MOT	6	0.55	0.88	0.86
Subscale	SEA	4	0.53	0.81	0.78
Subscale	OEA	4	0.51	0.80	0.76
Subscale	UOE	4	0.53	0.82	0.80
Subscale	ROE	4	0.55	0.83	0.80
Subscale	PE	5	0.51	0.83	0.81
Subscale	CI	4	0.52	0.81	0.77

Note. Validity indices at the subscale level (one-factor CFA with WLSMV, ordered items) and latent level (subscale indicators, ML). AVE = Average Variance Extracted; CR = Composite Reliability. Abbreviations: EI = Emotional Intelligence; SEA = Self-Emotion Appraisal; OEA = Others’ Emotion Appraisal; UOE = Use of Emotion; ROE = Regulation of Emotion; GRIT = L2 Grit; PE = Perseverance of Effort; CI = Consistency of Interest; MOT = L2 Motivation; MOT = Motivated Learning Behavior.

**Table 4:** Correlations Among Latent Constructs.

Construct	EI	GRIT	MOT	Performance
EI	1.00			
GRIT	0.43 ***	1.00		
MOT	0.55 ***	0.57 ***	1.00	
Performance	0.66 ***	0.63 ***	0.81 ***	1.00

Note. Pearson correlations among latent constructs (lower triangle). *** *p* < .001. Abbreviations: EI = Emotional Intelligence; SEA = Self-Emotion Appraisal; OEA = Others’ Emotion Appraisal; UOE = Use of Emotion; ROE = Regulation of Emotion; GRIT = L2 Grit; PE = Perseverance of Effort; CI = Consistency of Interest; MOT = L2 Motivation.

**Table 5:** Correlations Among Subscales.

Subscale	SEA	OEA	UOE	ROE	PE	CI	MOT
SEA	1.00						
OEA	0.52 ***	1.00					
UOE	0.49 ***	0.51 ***	1.00				
ROE	0.47 ***	0.52 ***	0.49 ***	1.00			
PE	0.36 ***	0.35 ***	0.30 ***	0.31 ***	1.00		
CI	0.25 ***	0.25 ***	0.27 ***	0.28 ***	0.53 ***	1.00	
MOT	0.48 ***	0.44 ***	0.40 ***	0.42 ***	0.57 ***	0.43 ***	1.00

Note. Pearson correlations among subscales (lower triangle). *** *p* < 0.001. Abbreviations: EI = Emotional Intelligence; SEA = Self-Emotion Appraisal; OEA = Others’ Emotion Appraisal; UOE = Use of Emotion; ROE = Regulation of Emotion; GRIT = L2 Grit; PE = Perseverance of Effort; CI = Consistency of Interest; MOT = L2 Motivation.

**Table 6:** Chain Mediation: Global Fit Indices.

Index	Value
χ^2^	302.11
Df	59
P	<0.001
CFI	0.953
TLI	0.937
RMSEA	0.072
RMSEA 90% CI	[0.064, 0.080]
SRMR	0.048

Note. χ^2^ = chi-square; df = degrees of freedom; CFI = Comparative Fit Index; TLI = Tucker–Lewis Index; RMSEA = Root Mean Square Error of Approximation (90% CI); SRMR = Standardized Root Mean Square Residual.

**Table 7:** Standardized Paths.

Outcome	Predictor	Beta	SE	Z	*p*	95% CI (Lower)	95% CI (Upper)
GRIT	EI	0.574	0.036	15.800	<.001	0.503	0.645
MOT	GRIT	0.515	0.043	11.919	<.001	0.430	0.599
MOT	EI	0.426	0.043	10.016	<.001	0.343	0.510
Performance	MOT	0.635	0.049	12.916	<.001	0.539	0.732
Performance	EI	0.185	0.034	5.432	<.001	0.118	0.251
Performance	GRIT	0.165	0.041	4.026	<.001	0.085	0.246

Note. Abbreviations: EI = Emotional Intelligence; SEA = Self-Emotion Appraisal; OEA = Others’ Emotion Appraisal; UOE = Use of Emotion; ROE = Regulation of Emotion; GRIT = L2 Grit; PE = Perseverance of Effort; CI = Consistency of Interest; MOT = L2 Motivation.

**Table 8:** Indirect Effects with Bootstrap Cis.

Effect	B_Std	SE_Std	z_Std	*p*_Std	CI_Low_Std	CI_High_Std
ind_EI_GRIT	0.095	0.025	3.781	<0.001	0.048	0.149
ind_EI_MOT	0.271	0.039	7.008	<0.001	0.199	0.350
ind_chain	0.188	0.026	7.326	<0.001	0.140	0.241
total_indirect	0.553	0.039	14.048	<0.001	0.476	0.628
total_effect_EI	0.738	0.033	22.423	<0.001	0.669	0.805

Note. Abbreviations: EI = Emotional Intelligence; SEA = Self-Emotion Appraisal; OEA = Others’ Emotion Appraisal; UOE = Use of Emotion; ROE = Regulation of Emotion; GRIT = L2 Grit; PE = Perseverance of Effort; CI = Consistency of Interest; MOT = L2 Motivation.

**Table 9:** SEM: R-squared.

Variable	*R* ^2^
Performance	0.787
GRIT	0.329
MOT	0.549

Note. *R*^2^ indicates the proportion of variance explained.

## Data Availability

The data presented in this study are available upon request from the corresponding author. The data are not publicly available because they consist of raw data that have been processed and analyzed for the purposes of this study.

## Appendix A

**Table A1:** 2nd-Order CFA (No Parcels): Standardized Loadings.

Factor	Indicator	Loading	SE	z	*p*
CI	CI4	0.779	0.031	17.325	<.001
CI	CI2	0.735	0.029	17.578	<.001
CI	CI3	0.716	0.031	16.007	<.001
CI	CI1	0.658	0.031	14.805	<.001
EI	SEA	0.851	0.155	10.504	<.001
EI	OEA	0.841	0.142	10.967	<.001
EI	ROE	0.763	0.094	12.508	<.001
EI	UOE	0.745	0.087	12.800	<.001
GRIT	PE	0.933	0.589	4.412	<.001
GRIT	CI	0.717	0.094	10.998	<.001
MOT	MOT5	0.883	0.017	53.420	<.001
MOT	MOT6	0.863	0.016	53.417	<.001
MOT	MOT3	0.695	0.025	28.076	<.001
MOT	MOT4	0.677	0.025	27.594	<.001
MOT	MOT1	0.643	0.026	24.988	<.001
MOT	MOT2	0.629	0.027	23.257	<.001
OEA	OEA2	0.938	0.039	12.857	<.001
OEA	OEA4	0.661	0.026	13.920	<.001
OEA	OEA1	0.632	0.024	14.085	<.001
OEA	OEA3	0.582	0.024	12.891	<.001
PE	PE3	0.933	0.068	4.926	<.001
PE	PE1	0.674	0.048	5.065	<.001
PE	PE5	0.672	0.048	5.084	<.001
PE	PE4	0.645	0.046	5.031	<.001
PE	PE2	0.581	0.043	4.830	<.001
ROE	ROE3	0.777	0.028	18.247	<.001
ROE	ROE4	0.733	0.027	17.424	<.001
ROE	ROE1	0.721	0.027	17.552	<.001
ROE	ROE2	0.719	0.027	17.495	<.001
SEA	SEA1	0.944	0.040	12.373	<.001
SEA	SEA2	0.677	0.026	13.499	<.001
SEA	SEA3	0.637	0.025	13.398	<.001
SEA	SEA4	0.591	0.025	12.373	<.001
UOE	UOE1	0.745	0.027	18.157	<.001
UOE	UOE3	0.736	0.026	19.093	<.001
UOE	UOE2	0.715	0.027	17.427	<.001
UOE	UOE4	0.711	0.026	18.119	<.001

Note. Standardized factor loadings (λ), standard errors (SE), z-values, and two-tailed *p*-values are reported.
